# The Efficacy and Safety of Inhaled Antibiotics for the Treatment of Bronchiectasis in Adults: Updated Systematic Review and Meta-Analysis

**DOI:** 10.1016/j.chest.2024.01.045

**Published:** 2024-02-02

**Authors:** Ricardo Cordeiro, Hayoung Choi, Charles S. Haworth, James D. Chalmers

**Affiliations:** a Department of Pulmonology, Centro Hospitalar do Oeste, Torres Vedras, Portugal; b Division of Pulmonary, Allergy, and Critical Care Medicine, Department of Internal Medicine, Hallym University Kangnam Sacred Heart Hospital, Hallym University College of Medicine, Seoul, South Korea; c Cambridge Centre for Lung Infection, Royal Papworth Hospital NHS Foundation Trust, University of Cambridge, Cambridge, England; d Department of Medicine, University of Cambridge, Cambridge, England; e Division of Molecular and Clinical Medicine, University of Dundee, Ninewells Hospital and Medical School, Dundee, Scotland

**Keywords:** antibiotics, bronchiectasis, inhalation, meta-analysis, therapeutics

## Abstract

**Background:**

Inhaled antibiotics are recommended conditionally by international bronchiectasis guidelines for the treatment of patients with bronchiectasis, but results of individual studies are inconsistent. A previous meta-analysis demonstrated promising results regarding the efficacy and safety of inhaled antibiotics in bronchiectasis. Subsequent publications have supplemented the existing body of evidence further in this area.

**Research Question:**

To what extent do inhaled antibiotics demonstrate both efficacy and safety as a treatment option for adults with bronchiectasis?

**Study Design and Methods:**

Systematic review and meta-analysis of randomized controlled trials of inhaled antibiotics in adult patients with bronchiectasis. We searched MEDLINE, Embase, the Cochrane Central Register of Controlled Trials, Web of Science, and ClinicalTrials.gov for eligible studies. Studies were included if they enrolled adults with bronchiectasis diagnosed by CT imaging and had a treatment duration of at least 4 weeks. The primary end point was exacerbation frequency, with additional key efficacy end points including severe exacerbations, bacterial load, symptoms, quality of life, and FEV_1_. Data were pooled through random-effects meta-analysis.

**Results:**

Twenty studies involving 3,468 patients were included. Inhaled antibiotics were associated with reduced number of patients with exacerbations (risk ratio, 0.85; 95% CI, 0.75-0.96), a slight reduction in exacerbation frequency (rate ratio [RR], 0.78; 95% CI, 0.68-0.91), a probable reduction in the frequency of severe exacerbations (RR, 0.48; 95% CI, 0.31-0.74), and a likely slight increase in time to first exacerbation (hazard ratio, 0.80; 95% CI, 0.68-0.94). Inhaled antibiotics likely lead to a slight increase in the Quality of Life Questionnaire-Bronchiectasis respiratory symptom score (mean difference, 2.51; 95% CI, 0.44-4.31) and may reduce scores on the St. George Respiratory Questionnaire (mean difference, –3.13; 95% CI, –5.93 to –0.32). Bacterial load consistently was reduced, but FEV_1_ was not changed with treatment. Evidence suggests little to no difference in adverse effects between groups (OR, 0.99; 95% CI, 0.75-1.30). Antibiotic-resistant organisms likely were increased by treatment.

**Interpretation:**

In this systematic review and meta-analysis, inhaled antibiotics resulted in a slight reduction in exacerbations, a probable reduction in severe exacerbations, and a likely slight improvement in symptoms and quality of life in adults with bronchiectasis.

**Trial Registry:**

International Prospective Register of Systematic Reviews; No.: CRD42023384694; URL: https://www.crd.york.ac.uk/prospero/.

**Take-home Points**

**Research Question:** How efficacious and safe are inhaled antibiotics in adults receiving treatment for bronchiectasis?

**Results:** In this systematic review and meta-analysis of 20 studies with 3,468 patients with bronchiectasis, inhaled antibiotics reduced the number of patients with exacerbations (rate ratio [RR], 0.85; 95% CI, 0.75-0.96), frequency of exacerbations (RR, 0.78; 95% CI, 0.68-0.91), frequency of severe exacerbations (RR, 0.48; 95% CI, 0.31-0.74), and prolonged time to first exacerbation (hazard ratio, 0.80; 95% CI, 0.68-0.94). Quality of life improved (St. George Respiratory Questionnaire: mean difference, –3.13; 95% CI, –5.93 to –0.32; Quality of Life Questionnaire-Bronchiectasis: mean difference, 2.51; 95% CI, 0.44-4.31). The adverse effects of inhaled antibiotics were comparable with those of placebo treatment (OR, 0.99; 95% CI, 0.75-1.30).

**Interpretation:** In adults with bronchiectasis, inhaled antibiotics were shown to reduce exacerbations and severe exacerbations and improve symptoms and quality of life.

Chronic infection with bacteria is a key component of the so-called vicious vortex of bronchiectasis. The most common organisms causing chronic infection in patients with bronchiectasis are *Pseudomonas aeruginosa* and *Haemophilus influenzae*, with *Streptococcus pneumoniae*, *Staphylococcus aureus*, *Moraxella catarrhalis*, and other enteric gram-negative organisms also being isolated frequently from sputum and BAL samples in these patients.^1^ Airway infection leads to chronic inflammation and impaired mucociliary clearance. Patients who are infected chronically therefore are at higher risk of recurrent exacerbations, and those infected with *P aeruginosa* are at particularly high risk. Studies suggest the presence of *P aeruginosa* is associated with an increase in exacerbations, a sevenfold increased risk of hospitalization, and threefold increased risk of mortality.^2^^,^^3^

Exacerbations are associated independently with impaired quality of life and mortality.^4^ Therefore, reducing the number of exacerbations is the cornerstone of long-term disease management. Long-term macrolides reduce exacerbations, including in patients with macrolide-tolerant organisms such as *P aeruginosa*.^5^, ^6^, ^7^ The use of inhaled antibiotics is an alternative that provides consistent deposition of high antibiotic concentrations directly to the site of infection with a lower risk of toxicity, systemic adverse events, and bacterial resistance.^8^^,^^9^ They have been part of the standard of care for patients with cystic fibrosis (CF) and have been in use for > 40 years in that patient population.^10^^,^^11^

The role of inhaled antibiotics in bronchiectasis unrelated to CF is less clear. Inhaled antibiotics currently are used in non-CF bronchiectasis management under distinct conditions: acute treatment for exacerbations, targeted eradication of *P aeruginosa*, and long-term maintenance therapy.^12^ The European Respiratory Society (ERS) guidelines in 2017 made a conditional recommendation to offer inhaled antibiotics to patients with *P aeruginosa* infection with ≥ 3 exacerbations/y, while recommending oral antibiotic prophylaxis with a macrolide such as azithromycin for patients without *P aeruginosa* infection.^12^ Subsequent to the ERS guidelines, a series of large phase 3 studies have increased the evidence base substantially for inhaled antibiotics in patients with bronchiectasis. A systematic review and meta-analysis conducted in 2019 including the results of the large RESPIRE and ORBIT trials concluded that inhaled antibiotics achieve a small but significant decrease in exacerbations compared with placebo without improvements in symptoms or quality of life.^13^ Subsequent publications have challenged this, suggesting an improvement in symptoms (cough and sputum production) when bacterial load is reduced with inhaled antibiotics.^14^

The inconsistent results achieved in individual trials leave a series of unanswered questions for clinicians regarding inhaled antibiotics, including the magnitude of potential benefits on exacerbations, whether patients should expect improvements in symptoms and quality of life with inhaled antibiotics, and whether benefits are limited only to patients with *P aeruginosa* infection or also extend to infection with other organisms. To address these questions, we performed an updated systematic review and meta-analysis of inhaled antibiotic use in adults with bronchiectasis.

## Study Design and Methods

### Search Strategy and Selection Criteria

We report an update of the previously conducted meta-analysis published in 2019.^13^ The previous study searched relevant dates from inception through January 2019. To update the search, two investigators searched PubMed/MEDLINE, EMBASE, and the Cochrane Central Register of Controlled Trials from January 21, 2019, through December 13, 2022, for randomized controlled trials on long-term use of inhaled antibiotics in adult patients with bronchiectasis and chronic respiratory infections. For this review, the recently updated Preferred Reporting Items for Systematic Reviews and Meta-Analyses guidelines were used.^15^ No language restrictions were applied.

Studies were considered eligible for review if they included adult patients (aged ≥ 18 years) with CT scan-confirmed bronchiectasis, used inhaled antibiotics as a treatment for stable patients (defined by the absence of exacerbation at baseline), had a duration of at least 4 weeks, and measured at least one of the prespecified clinical outcomes. The intervention group included any antibiotic class given through inhalation. The control group could be those receiving inhaled placebo (eg, saline solution) or no therapy.

We excluded trials that included patients with bronchiectasis resulting from CF, enrolled patients younger than 18 years, and administered treatment exclusively during an acute exacerbation of bronchiectasis. We also excluded nonrandomized trials and observational studies. The search criteria were applied in two stages. First, clearly ineligible studies were excluded based on abstract review only. Second, full manuscript review was used to determine final eligibility.

Unpublished work was identified by searching for the 2019 through 2022 abstract books of the largest respiratory medicine conferences, the American Thoracic Society conference and the ERS conference. Studies were included for review if they complied with the aforementioned inclusion criteria. Additionally, we searched the ClinicalTrials.gov registry using the term *bronchiectasis* as a query. To supplement these searches, the reference lists of relevant publications, previous meta-analyses, and guidelines were reviewed.

Two investigators (R. C. and H. C.) independently performed the study selection, reviewing all the citations and abstracts identified to assess which articles would be included. Disagreements were resolved by consensus discussion. If an agreement was not reached, a third investigator (J. D. C.) was available to review the article. We assessed risk-of-bias using the Cochrane risk-of-bias tool, RoB 2, as per Cochrane suggestions on updated meta-analyses.

### Outcomes

End points were prespecified based on the previous meta-analysis and expert consensus on outcomes selected as important or critical in the European bronchiectasis guidelines.^12^ The primary outcome was the frequency of exacerbations. Additional selected outcomes included time to first exacerbation, proportion of patients with at least one exacerbation, frequency of severe exacerbations, quality of life (measured with the quality-of-life bronchiectasis questionnaire or the St. George Respiratory Questionnaire), lung function (measured as change in FEV_1_ % predicted), sputum bacterial load (measured as change in colony-forming units/g, 24-h sputum volume), and percentage of sputum cultures with negative results.^16^^,^^17^ Adherence and mortality also were evaluated. Outcome data selection was based on values reported at the end of the intervention.

Safety end points were assessed by collecting data on the number of patients with treatment-emergent adverse events (TEAEs), the number of patients with adverse events that led to discontinuation, treatment-emergent serious adverse events, and the number of patients with bronchospasm as an adverse event of special interest. Bacterial resistance in sputum, defined as the proportion of bacterial isolates with a minimum inhibitory concentration of more than the resistance threshold, was also collected.

Planned subgroup analyses included antibiotic agent (aminoglycosides, fluoroquinolones, β-lactams, and polymyxins) and baseline infection status (populations limited exclusively to patients infected with *P aeruginosa* vs populations with mixed pathogens or no prespecified pathogen selection).

### Data Analysis

Two authors (R. C. and H. C.) extracted end points of interest in a masked fashion. Data from each study were tabulated using a predesigned spreadsheet before inclusion in the analysis. For categorical binary outcomes, data with the number of participants with each outcome event were assessed in both the intervention and the placebo groups. Wherever possible, the intention-to-treat population was used as the denominator. For continuous outcomes, sample size, mean, SD, SE, or median (interquartile range) were extracted. If the mean relative change from baseline for each group and the SD or SE were reported, those data also were extracted. If not present, we calculated the mean difference and the 95% CIs for estimated pooled treatment effect, according to the recommended methods from the Cochrane Collaboration.^18^ If continuous outcomes were reported using different units or scales, a standardized mean difference and 95% CIs were calculated. For time-to-event outcomes, data were obtained from Cox proportional hazards model estimates when the log hazard ratio (HR) and its SE were provided by the study authors. Raw unadjusted data were sought wherever feasible. However, if the only available data were from adjusted models, these were pooled for analysis. For effect estimates, we obtained the number of participants in each group, the magnitude of the effect, and the respective CIs.

For the new studies, two authors (R. C. and H. C.) independently assessed the risk of bias by using Cochrane’s collaboration revised risk-of-bias tool present in RevMan software version 5.4.1. A third author (C. S. H.) resolved any discordant data. Each potential source of bias was graded as low risk, unclear risk, or high risk of bias.

We expected high clinical heterogeneity because of evaluating different interventions (type of antibiotic used, difference in inhaler devices, dosage regimens) and differences in inclusion and exclusion criteria. Therefore, data were pooled using a random effects model. We performed analyses both with and without outlying studies as part of a sensitivity analysis.^18^ When data had been estimated, sensitivity analyses excluding such data were performed to check the influence of any assumptions on the reported pooled effects. The *P* value from the χ^2^ test and the *I*^2^ CIs are provided to describe heterogeneity. We considered substantial heterogeneity when the *I*^2^ was ≥ 50%.^18^ We evaluated certainty of evidence by using the Grading of Recommendations, Assessment, Development, and Evaluations method for the selected outcomes. Meta-analyses were carried out using RevMan version 5.4.1 software. The review protocol was registered prospectively with the International Prospective Register of Systematic Reviews (identifier, CRD42023384694). More detailed information on the study methods is provided in e-Appendix 1.

## Results

**Figure 1:**
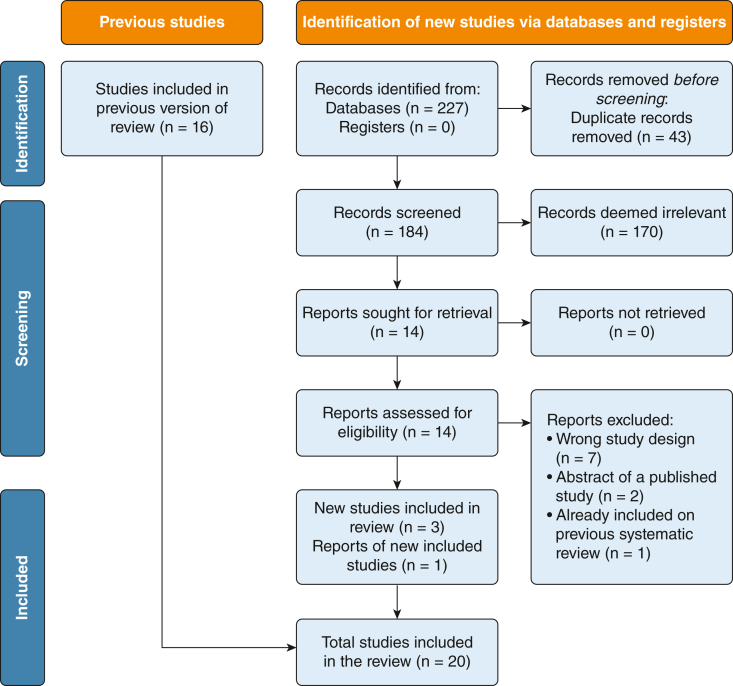
Preferred Reporting Items for Systematic Reviews and Meta-Analyses study flow diagram.

The updated search identified 227 references. After the removal of 43 duplicates, 184 studies were screened. From these, 170 studies were deemed irrelevant based on title and abstract. One study was identified as an abstract and presentation at the ERS conference.^19^ We assessed 14 studies for eligibility. Ten studies were excluded. We included the four remaining studies alongside the 16 trials included in the previous meta-analysis, resulting in a total of 20 trials (Fig 1).^19^, ^20^, ^21^, ^22^, ^23^, ^24^, ^25^, ^26^, ^27^, ^28^, ^29^, ^30^, ^31^, ^32^, ^33^ Of the four newly identified trials, two studies compared inhaled tobramycin with inhaled placebo, one using a conventional compressor nebulizer and the other using a vibrating mesh nebulizer.^27^^,^^33^ One study compared dry powder tobramycin with a placebo.^26^ The final study compared inhaled colistimethate sodium with placebo through an adaptive aerosol delivery mesh nebulizer.^19^ The primary end points of new studies were the number of exacerbations (Bronchiectasis and Tobramycin Solution Inhalation Therapy [BATTLE] trial), changes from baseline in *P aeruginosa* sputum density (i-BEST and Tobramycin in Bronchiectasis Colonized With Pseudmonas AeruginosaI [TORNASOL] studies), and exacerbation rate (PROMIS-I).^19^^,^^27^^,^^33^

In the previously identified 16 studies, eight assessed inhaled antibiotics in patients with *P aeruginosa* infection, and the remaining included patients infected with *P aeruginosa*, other pathogens, or a combination. Three new studies included only patients with *P aeruginosa*, and one study included other pathogens.^33^ Most studies included bronchiectasis populations of predominantly female patients with a mean age of 53 to 70 years. Study duration ranged from 6 weeks to 15 months, with treatment periods ranging from 4 weeks to 52 weeks. The 20 trials included reported data from 3,468 independent participants. The number of participants ranged from 15 to 304 patients (Table 1).^19^, ^20^, ^21^, ^22^, ^23^, ^24^, ^25^, ^26^, ^27^, ^28^, ^29^, ^30^, ^31^, ^32^, ^33^

**Table 1:** Characteristics of the Included Studies

Study	Drug	Duration	Intervention vs Control Groups					Primary Outcome	Secondary outcomes
			No. of Participants	Age, y	FEV_1_ % Predicted	*P aeruginosa* Present	Other Pathogens Present		
Aksamit et al (2018)^21^ (RESPIRE 2-28 d^a^)	Ciprofloxacin DPI (32.5 mg) vs placebo twice/d	12 mo	171 (92 female, 79 male) vs 86 (52 female, 34 male)	59.3 ± 14.2 vs 60.6 ± 13.7	56.4 ± 18.8 vs 56.2 ± 18.2	99 (58) vs 54 (63)	≥ 1 prespecified microorganism for recruitment: *Haemophilus influenzae*, *Moraxella catarrhalis*, *S aureus*, *S pneumoniae*, *Stenotrophomonas maltophilia*, *Burkholderia cepacia*	Time to first exacerbation, frequency of exacerbations	Less stringent definition of an exacerbation microbiologic outcomes, QOL assessments (SGRQ and QOL-B), lung function
Aksamit et al (2019)^21^ (RESPIRE 2-14 d)^a^	Ciprofloxacin DPI (32.5 mg) vs placebo twice/d	12 mo	176 (96 female, 80 male) vs 88 (62 female, 26 male)	60.4 ± 13.7 vs 60.4 ± 15.0	54.3 ± 17.3 vs 55.8 ± 18.6	107 (61) vs 55 (63)	≥ 1 prespecified microorganism for recruitment: *H influenzae*, *M catarrhalis*, *S aureus*, *S pneumoniae*, *S maltophilia*, *B cepacia*	Time to first exacerbation, frequency of exacerbations	Less stringent definition of an exacerbation microbiologic outcomes, QOL assessments (SGRQ and QOL-B), lung function
Barker et al (2000)^32^	Nebulized tobramycin (300 mg) vs placebo (1.25 mg quinine in saline) twice/d	6 wk	37 (23 female, 14 male) vs 37 (22 female, 15 male)	66.6 ± 13.0 vs 63.2 ± 13.5	56.2 ± 21.2 vs 53.3 ± 22.1	37 (100) vs 37 (100)	No data	Change in *P aeruginosa* density (CFU/g) from baseline to wk 4	Change in *P aeruginosa* density from baseline to wk 2 and to wk 6, investigator’s subjective assessment of change in patient general medical condition, percentage change in FEV_1_ and FVC % predicted, and safety measurements
Barker et al (2014)^29^ (AIR-BX 1)	Nebulized aztreonam (75 mg) vs placebo tid	28 wk	134 (84 female, 50 male) vs 132 (97 female, 35 male)	64.2 ± 12.9 vs 64.9 ± 12.1	60.4 ± 22.6 vs 64.5 ± 18.7	112 (84) vs 105 (80)	History of NTM: 16 (12) vs 14 (10); no data for other organisms	Change in QOL-B RSS score from baseline to wk 4	Change in QOL-B RSS score from baseline to wk 12, time to first exacerbation by wk 16, change in CFU/g, presence or absence of respiratory pathogens, changes in MIC of aztreonam
Barker et al (2014)^29^ (AIR-BX 2)	Nebulized aztreonam (75 mg) vs placebo tid	28 wk	136 (89 female, 47 male) vs 138 (101 female, 37 male)	63.3 ± 14.2 vs 62.7 ± 13.3	63.8 ± 19.5 vs 63.4 ± 13.3	116 (85) vs 103 (75)	History of NTM: 8 (6) vs 12 (9); no data for other organisms	Change in QOL-B RSS score from baseline to wk 4	Change in QOL-B RSS score from baseline to wk 12, time to first exacerbation by wk 16, change in CFU/g, presence or absence of respiratory pathogens, changes in MIC of aztreonam
De Soyza et al (2018)^20^ (RESPIRE 1-28 d)^a^	Ciprofloxacin DPI (32.5 mg) vs placebo twice/d	12 mo	141 (101 female and 40 male) vs 70 (52 female and 18 male)	64.2 ± 12.1 vs 64 ± 13.5	59.48 ± 15.1 vs 61.7 ± 16.7	83 (59) vs 45 (64)	≥ 1 prespecified microorganism for recruitment: *H influenzae*, *M catarrhalis*, *S aureus*, *S pneumoniae*, *S maltophilia*, *B cepacia*	Time to first exacerbation, frequency of exacerbations	Less stringent definition of an exacerbation microbiologic outcomes, QOL assessments (SGRQ and QOL-B), lung function
De Soyza et al (2018)^20^ (RESPIRE 1-14 d)^a^	Ciprofloxacin DPI (32.5 mg) vs placebo twice/d	12 mo	137 (88 female and 49 male) vs 68 (44 female and 24 male)	65.2 ± 13.5 vs 65.5 ± 12.9	59.42 ± 16.7 vs 57.37 ± 15.5	83 (61) vs 41 (64)	≥ 1 prespecified microorganism for recruitment: *H influenzae*, *M catarrhalis*, *S aureus*, *S pneumoniae*, *S maltophilia*, *B cepacia*	Time to first exacerbation, frequency of exacerbations	Less stringent definition of an exacerbation microbiologic outcomes, QOL assessments (SGRQ and QOL-B), lung function
Drobnic et al (2005)^25^	Nebulized tobramycin (300 mg) vs placebo (0.9% saline) twice/d; crossover trial	13 mo	10 vs 10 in the PP population of 30 participants included in the ITT population (sex breakdown not reported)	Mean, 64.5 (range, 38-75)	51.78 ± 16.5	10 (100) vs 10 (100)	No data	Not specifically stated, but presumed to be no. of exacerbations	No. of hospital admissions, No. of hospital admission days, antibiotic use, pulmonary function, SGRQ score, tobramycin toxicity, density of *P aeruginosa* in sputum, emergence of bacterial resistance, and emergence of other opportunistic bacteria
Guan et al (2023)^27^^b^	Nebulized TIS 300 mg vs placebo (normal saline) twice/d	16 wk	167 (109 female and vs 58 male) and 172 (112 female and 60 male)	53.0 ± 13.0 vs 54.0 ± 12.0	60.9 ± 21.5 vs 63.6 ± 22.5	167 (100) vs 172 (100)	No data	Change from baseline in *P aeruginosa* density on d 29; QOL-B RSS score change from baseline	Time to the first exacerbation, frequency of exacerbations, rate of negative culture results on d 29, 24-h sputum volume and sputum purulence, change from baseline in BHQ, MIC on d 29 and 85, tobramycin blood concentration on d 1 and 28.
Haworth et al (2014)^28^	Nebulized colistin (1 million International Units) vs placebo (0.45% saline) twice/d	26 wk	73 (46 female, 27 male) vs 71 (37 female, 34 male)	58.3 ± 15.3 vs 60.3 ± 15.8	55.9 ± 24.3 vs 57.6 ± 24.9	73 (100) vs 71 (100)	*H influenzae*: 0 vs 1 (1%); *S aureus*: 3 (4%) vs 5 (7%); *S pneumoniae*: 2 (3%) vs 0; *S maltophilia*: 0 vs 0; *M catarrhalis*: 3 (4%) vs 2 (3%)	Time to exacerbation	Time to exacerbation (based on adherence recorded by the I-neb), severity of exacerbation, CFUs of *P aeruginosa*, 24-h sputum weight, SGRQ score, bronchoconstriction in 30 min after first dose of study drug, FEV_1_, sensitivity of *P aeruginosa* to colistin, CFUs of other potentially pathogenic organisms, and adverse event reporting
Haworth et al (2019)^22^ (ORBIT-3)	Liposomal ciprofloxacin (liposome encased ciprofloxacin [135 mg] and free ciprofloxacin [54 mg]) vs placebo (empty liposomes in 0.9% saline) once daily	48 wk	183 (127 female, 56 male) vs 95 (67 female, 28 male)	64.3 ± 13.6 vs 66.7 ± 10.7	57.3 ± 21.9 vs 57.4 ± 20.2	183 (100) vs 95 (100)	*S aureus*: 31 (17%) vs 22 (23%); *Escherichia coli* and coliforms: 11 (6%) vs 5 (5%); *S pneumoniae*: 5 (3%) vs 3 (3%); *H influenzae*: 5 (3%) vs 1 (1%); *M catarrhalis*: 2 (1%) vs 0	Time to first pulmonary exacerbation	No. and frequency of pulmonary exacerbations, no. of patients requiring IV antibiotics, QOL-B RSS score, change in *P aeruginosa* bacterial density (CFU/g)
Haworth et al (2019)^22^ (ORBIT-4)	Liposomal ciprofloxacin (liposome encased ciprofloxacin [135 mg] and free ciprofloxacin [54 mg]) vs placebo (empty liposomes in 0.9% saline) once daily	48 wk	206 (134 female, 72 male) vs 98 (63 female, 35 male)	63.3 ± 13.6 ± vs 64.2 ± 12.6	62.6 ± 22.2 vs 59.8 ± 20.8	206 (100) vs 98 (100)	*S aureus*: 50 (24%) vs 23 (24%); *E coli* and coliforms: 9 (4%) vs 3 (3%); *S pneumoniae*: 10 (5%) vs 3 (3%); *H influenzae*: 7 (3%) vs 4 (4%)	Time to first pulmonary exacerbation	No. and frequency of pulmonary exacerbations, No. of patients requiring IV antibiotics, QOL-B RSS score, change in *P aeruginosa* bacterial density (CFU/g)
Haworth et al (2021)^19^^b^	Colistimethate sodium (CMS I-neb) vs placebo I-neb (0.45% saline) twice/d	12 mo	176 (123 female and 53 male) vs 197 (126 female and 71 male)	64.2 ± 14.9 vs 64.2 ± 13.1	62.4 ± 20.7 vs 64.5 ± 18.9	176 (100) vs 197 (100)	No data	Mean annual exacerbation rate	Mean annual severe exacerbation rate, time to first exacerbation, change in SGRQ total score, change in *P aeruginosa* sputum density, *P aeruginosa* resistance to colistimethate sodium
Loebinger et al (2021)^26^^b^	TIP in 3 cohorts with 2 intervention groups (cyclical vs continuous; 84 mg, 140 mg, or 224 mg daily) vs placebo	112 d	86 (33 male and 53 female) vs 21 (8 male and 13 female)	62.52 ± 14.12 vs 67.23 ± 11.00	59.71 ± 21.52 vs 59.50 ± 18.24	86 (100) vs 21 (100)	No data	Change from baseline to d 29 in *P aeruginosa* density in sputum	Antimicrobial efficacy of TIP, effect of different doses of TIP and different regimens on pulmonary exacerbations, efficacy profile of different doses of TIP and different regimens as measured by antipseudomonal antibiotic use
Murray et al (2011)^24^	Nebulized gentamicin (80 mg) vs placebo (0.9% saline) twice/d	15 mo	2 vs 33 randomly assigned; 27 (18 female, 9 male) vs 30 (15 female, 15 male) completed and included in the analysis	Median (IQR), 58 (53-67) vs 64 (56-69)	median (IQR): 72.9 (60-81.2) vs 63.4 (45.5-80.4)	13 (48) vs 11 (37)	*H influenzae*: 11 (41%) vs 15 (50%); *S aureus*: 2 (7%) vs 1 (3%); *S pneumoniae*: 1 (4%) vs 0; *M catarrhalis*: 0 vs 2 (7%); coliforms: 0 vs 1 (3%)	Quantitative bacteriology in CFU/g	Sputum purulence and 24-h volume, pulmonary function test results, exercise capacity, LCQ and SGRQ scores, exacerbation frequency, inflammatory biomarkers
Orriols et al (1999)^30^	Nebulized ceftazidime (1,000 mg) and tobramycin (100 mg) twice/d vs standard care	12 mo	7 (1 female, 6 male) vs 8 (4 female, 4 male)	Mean, 62.0 (SEM 8.5) vs 61.4 (10.3)	62.3 (SEM 19.9) vs 56.2 (21.4)	7 (100) vs 8 (100)	No data	Not specifically stated, but presumed to be no. of hospital admissions	Length of hospitalization (d), use of oral antibiotics, FVC, FEV_1_, Pao_2_, Paco_2_, drug toxicity, and emergence of bacterial resistance
Serisier et al (2013)^23^ (ORBIT-2)	Liposomal ciprofloxacin (liposome encased ciprofloxacin [135 mg] and free ciprofloxacin [54 mg]) vs placebo (empty liposomes in 0.9% saline) once daily	24 wk	20 (10 female, 10 male) vs 22 (13 female, 9 male)	70 ± 5.6 vs 59.5 ± 13.2	60.7 ± 24.1 vs 53.1 ± 22.7	20 (100) vs 22 (100)	*Klebsiella*: 2 (10%) vs 2 (9%); *Ochrobactrum anthropic*: 0 vs 2 (9%)	Mean change in sputum *P aeruginosa* bacterial density (CFU/g) from baseline to end of first treatment cycle (28 d)	Time to first pulmonary exacerbation, FEV_1_, 6MWT, SGRQ score, safety, and tolerability
Terpstra et al (2022)^33^^b^	Nebulized TIS 300 mg vs placebo (NaCl 0.9%) once daily	52 wk	26 (13 female and 13 male) vs 26 (17 female and 9 male)	67.9 ± 6.6 vs 64.1 ± 14.0	65.9 ± 24.9 vs 70.5 ± 24.0	5 (19.2) vs 9 (34.6)	*H influenzae*: 7 (26.9) vs 9 (34.6); *S aureus*: 4 (15.4%) vs 2 (7.7%); *S pneumoniae* 0 (0%) vs 1 (3.8%); other: 10 (38.4%) vs 5 (19.3%)	No. of exacerbations during the 1-y treatment period	Time to next exacerbation, change in lung function, change in QOL measurements based on LRTI-VAS, QOL-B and the LCQ score
TR02-107 (NCT00775138)^34^	Nebulized liposomal amikacin (280 mg and 560 mg) vs placebo (empty liposomes in 1.5% saline) once daily	56 d	24 (10 female, 14 male) vs 19 (11 female, 8 male) vs 19 (9 female, 10 male)^a^	49.9 ± 21.1 vs 58.5 ± 16.0 vs 49.4 ± 13.3^a^	64.5 ± 20.7 vs 71.4 ± 23.9 vs 62.6 ± 15.7^a^	24 (100) vs 19 (100) vs 19 (100)^a^	No data	Safety of liposomal amikacin as measured by proportion of adverse events, change in oxygen saturations, change in FEV_1_	Frequency of cough with expectoration, PSSS, SGRQ score, bacterial density of *P aeruginosa* (CFU/g), pulmonary exacerbations
Wilson et al (2013)^31^	Ciprofloxacin DPI (32.5 mg) vs placebo twice/d	84 d	60 (39 female and 21 male) vs 64 (43 female and 21 male)	64.7 ± 11.8 vs 61.4 ± 11.9	57.2 ± 13.7 vs 54.6 ± 14.8	32 (53) vs 35 (55)	*H influenzae*: 14 (23%) vs 16 (25%); *S aureus*: 8 (13%) vs 17 (27%); *S pneumoniae*: 7 (12%) vs 2 (3%); *M catarrhalis*: 5 (8%) vs 3 (5%)	Effect of ciprofloxacin DPI on total bacterial density of predefined potential respiratory pathogens in sputum (CFU/g) after the 28-d treatment period	Time to exacerbation; emergence of new potential respiratory pathogens; emergence of resistance among baseline pathogens; changes in inflammatory biomarkers; change in 24-h sputum volume and color; changes in FEV_1_, FVC, and SGRQ score at d 29, 56, and 84; adverse events; results of physical examinations; vital signs; and laboratory analyses

Data are No. (%) or mean ± SD, unless otherwise specified. 6MWT = 6-min walk test; BHQ = Bronchiectasis Health Questionnaire; CFU = colony-forming units; CMS = colistimethate sodium; DPI = dry powder for inhalation; IQR = interquartile range; ITT = intention-to-treat; LCQ = Leicester Cough Questionnaire; LRTI-VAS = lower respiratory tract infections visual analog scale; MIC = minimum inhibitory concentration; NTM = nontuberculous mycobacterial infection; QOL = quality of life; QOL-B = Quality of Life Questionnaire-Bronchiectasis; PP = per protocol; PSSS = pulmonary symptom severity score; RSS = respiratory symptoms scale; SEM = standard error of the mean; SGRQ = St. George Respiratory Questionnaire; TIP = tobramycin inhalation powder; TIS = tobramycin inhalation solution.

a 280 mg group vs 560 mg group vs placebo group.

b Trials not reported in the previous meta-analysis. The RESPIRE-1 and RESPIRE-2 trials underwent assessment as distinct studies for the 14-d and 28-d cohorts; however, a pooled placebo group was used as a comparator.

### Outcomes of Included Studies

#### Exacerbations

Trials assessed reported multiple exacerbation end points, expressing them as frequency of exacerbations, frequency of severe exacerbations, time to first exacerbation, and proportion of patients with at least one exacerbation. The number of patients experiencing at least one exacerbation was reported in 17 studies.^20^, ^21^, ^22^, ^23^, ^24^^,^^26^, ^27^, ^28^, ^29^^,^^31^, ^32^, ^33^, ^34^ In the intervention group, 714 of 1,823 patients (39.1%) reported at least one exacerbation compared with 522 of 1,237 patients (42.1%) in the control group. The pooled relative risk was 0.85 (95% CI, 0.76-0.96; *P* = .006) with nonsubstantial heterogeneity (*I*^2^ = 40%) and no significant subgroup difference between classes of antibiotics (*P* = .26). These data indicate that inhaled antibiotic treatment is associated with a significant reduction in the proportion of patients experiencing at least one exacerbation.

Frequency of exacerbations expressed as a rate during trial follow-up was reported in 12 trials (N = 2,930).^19^, ^20^, ^21^, ^22^^,^^26^^,^^27^^,^^29^^,^^33^ Our meta-analysis confirmed that inhaled antibiotics significantly reduced exacerbation frequency (rate ratio [RR], 0.79; 95% CI, 0.68-0.91; *P* = .0009), with moderate heterogeneity (*I*^2^ = 42%). Subgroup analysis revealed a nonsignificant increase in exacerbation rate with aztreonam (RR, 1.13; 95% CI, 0.84-1.52; *P* = .44; *I*^2^ = 0). Pooled analysis of fluoroquinolones, colistin, and aminoglycosides showed a significant decrease in exacerbation rate (RR, 0.73; 95% CI, 0.65-0.84; *P* < .00001), with low heterogeneity (*I*^2^ = 20%).

**Figure 2:**
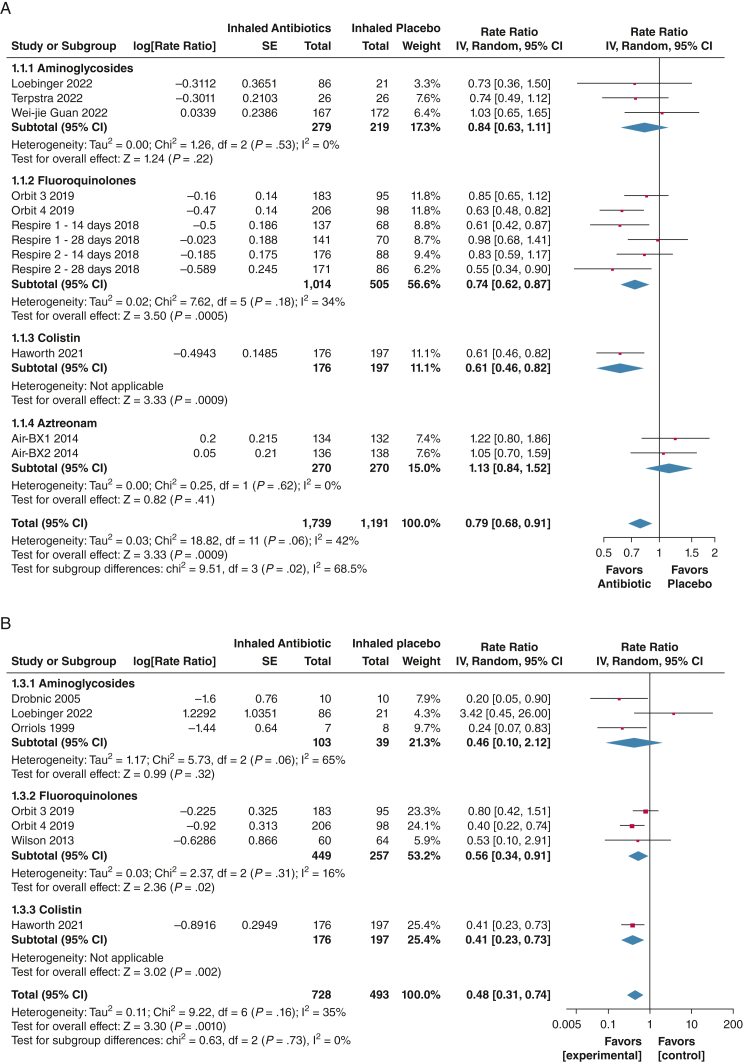
A, B, Forest plots showing frequency of exacerbations (A) and frequency of severe exacerbations (B). The weight of each study is the percentage of its contribution to the overall effect estimate. Weights of individual studies might not add up to the subtotal or overall weights because of rounding. Risk of bias is represented as low (+), unclear (?), or high (–). df = degrees of freedom; IV = inverse variance.

Frequency of severe exacerbations (defined as exacerbation resulting in hospitalization or IV antibiotics) expressed as a rate during the trial follow-up were reported in seven trials (N = 1,221).^22^^,^^25^^,^^26^^,^^30^^,^^31^ Pooled analysis showed significant reduction of severe exacerbations with inhaled antibiotic treatment (RR, 0.48; 95% CI, 0.31-0.74; *P* = .0010; *I*^2^=35%), with no subgroup differences (*P* = .73; *I*^2^ = 0%) (Fig 2).

Time to first exacerbation was reported in an extractable format in the 12 trials (N = 2,752).^19^, ^20^, ^21^, ^22^, ^23^^,^^27^, ^28^, ^29^^,^^33^ Inhaled antibiotics prolonged the time to first exacerbation (HR, 0.80; 95% CI, 0.68-0.94; *P* < .0001), with moderate heterogeneity (*I*^2^ = 45%). Subgroup analysis showed no significant subgroup difference among aminoglycosides, fluoroquinolones, and colistin (*I*^2^ = 6.9%; *P* = .34). Decreased time to first exacerbation was found with aztreonam (HR, 1.25; 95% CI, 0.91-1.71; *P* = .17; *I*^2^ = 0%).

### Symptoms and Quality of Life

Inhaled antibiotics were associated with a significant improvement in respiratory symptoms using the Quality of Life Questionnaire-Bronchiectasis in 11 trials (N = 2,315), with a mean difference change from baseline of 2.37 points (95% CI, 0.44-4.31; *P* = .04; *I*^2^ = 48%), which is lower than the minimal clinically important difference of 8 points.^16^ Only one study showed an average improvement of a > 8-point difference in the per-protocol population.^27^

**Figure 3:**
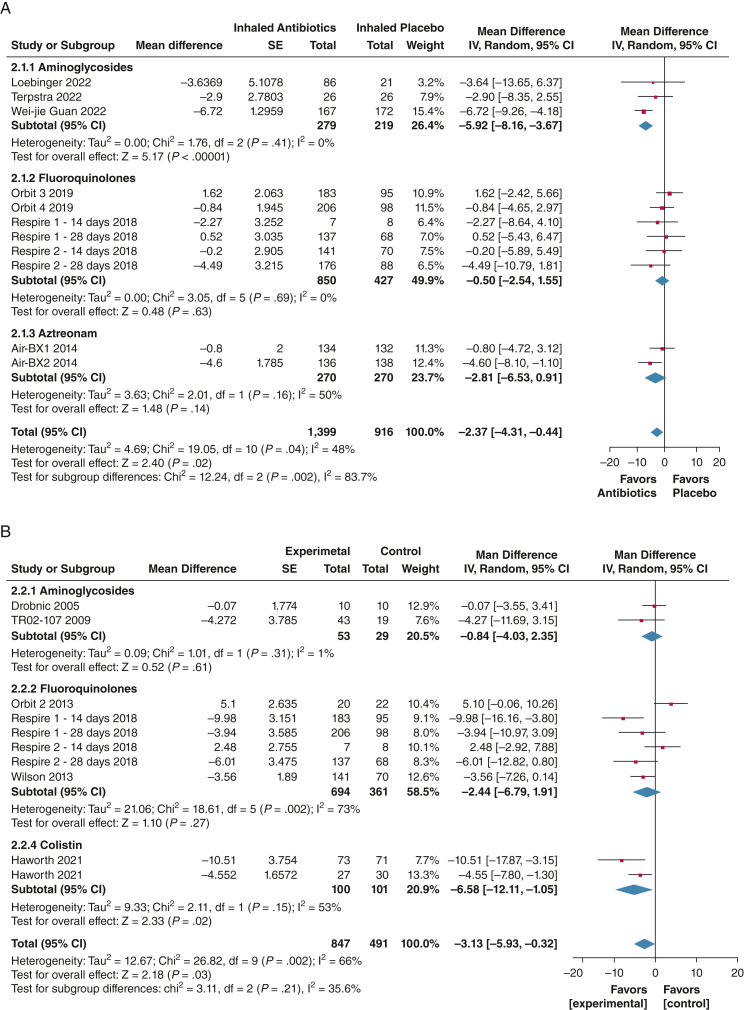
A, B, Forest plots showing of quality of life and symptom scales according to the Quality of Life Questionnaire-Bronchiectasis (QOL-B) (A) and the St. George Respiratory Questionnaire (SGRQ) (B). A negative score has been shown as a reduction in symptoms for ease of interpretation. In the original scales, a reduction in the scale indicates an improvement in symptoms with the SGRQ, but a worsening with the QOL-B. The weight of each study is the percentage of its contribution to the overall effect estimate. Weights of individual studies might not add up to the subtotal or overall weights because of rounding. Risk of bias is represented as low (+), unclear (?), or high (–). df = degrees of freedom; IV = inverse variance; M-H = Mantel-Haenszel.

For the St. George Respiratory Questionnaire, pooled analysis of 10 trials (N = 1,338) showed a significant difference in favor of the intervention (mean difference, –3.13; 95% CI, –5.93 to –0.32; *P* = .03), with substantial heterogeneity (*I*^2^ = 66%).^19^, ^20^, ^21^^,^^23^^,^^25^^,^^28^^,^^31^ The average between-group different was lower than the minimal clinically important difference of 4 points. Subgroup analysis of inhaled colistin showed a mean difference of more than the 4-point minimal clinically important difference (–6.58; 95% CI, –12.11 to –1.05; *P* = .002; *I*^2^ = 66%) (Fig 3).^19^^,^^28^

Sputum volume assessed as 24-h sputum volume in milliliters was reported previously in three trials (N = 540).^24^^,^^27^^,^^28^ The pooled mean difference was –4.63 mL/24 h (95% CI, –8.42 to –0.84; *P* = .02; *I*^2^ = 0%).

### Lung Function

A nonsignificant mean deterioration in FEV_1_ of –0.91% (95% CI, –2.01 to 0.19; *P* = .10; *I*^2^ = 0%) was estimated from nine trials (N = 1,437).^22^^,^^24^^,^^25^^,^^29^^,^^31^^,^^33^^,^^34^ Other related outcomes present at previous meta-analysis (absolute FEV_1_ change or 6-min walking test distance) were not updated because no new data were identified.

### Antibiotic Resistance

**Figure 4:**
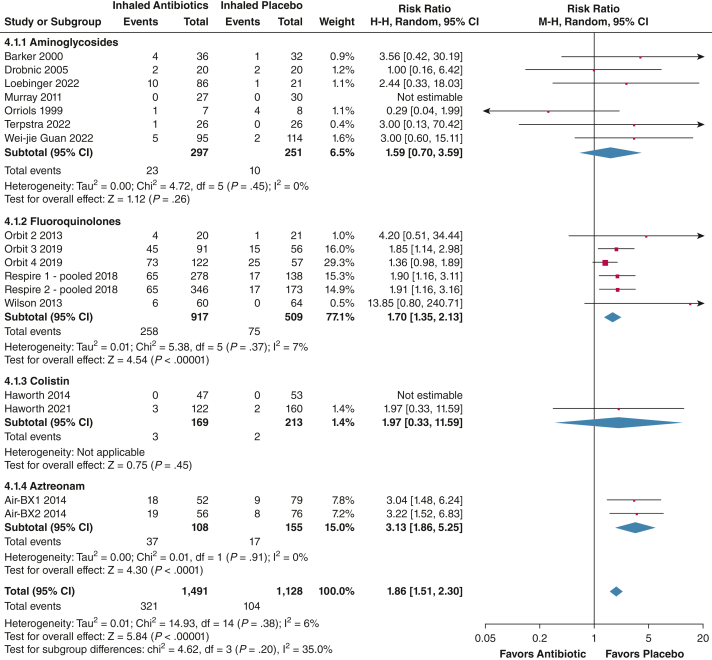
Forest plot showing isolated bacteria with a minimum inhibitory concentration of more than the resistant breakpoint at the end of treatment. The weight of each study is the percentage of its contribution to the overall effect estimate. Weights of individual studies might not add up to the subtotal or overall weights because of rounding. Risk of bias is represented as low (+), unclear (?), or high (–). df = degrees of freedom; IV = inverse variance.

The pooled risk ratio of isolating a resistant organism was 1.86 (95% CI, 1.51-2.30; *P* < .00001), with low heterogeneity (*I*^2^ = 6%), indicating a higher risk of resistance with inhaled antibiotic treatment (see e-Tables 1 and 2 for the definition of emergent resistance). Resistance increased regardless of which inhaled antibiotic was used (*P* = .20; *I*^2^ = 35%). The number of patients with resistant organisms at the end of treatment in the intervention groups was 321 patients (21.5%) and 104 patients (9.2%) in the placebo groups, with data from 19 trials (N = 2,619) (Fig 4).^19^, ^20^, ^21^, ^22^, ^23^, ^24^, ^25^, ^26^, ^27^, ^28^, ^29^, ^30^, ^31^, ^32^, ^33^

### Safety

Fifteen studies reported data for TEAEs.^19^, ^20^, ^21^, ^22^, ^23^^,^^25^^,^^27^, ^28^, ^29^^,^^31^^,^^32^ The proportion of patients with TEAE was 79.2% (1,461/1,844) in the intervention group and 79.8% (1,088/1,363) in the placebo group (OR, 0.99; 95% CI, 0.75-1.30; *P* = .94; *I*^2^ = 42%). Subgroup analysis showed increased odds of experiencing at least one adverse event with aztreonam (OR, 2.13; 95% CI, 1.16-3.93; *P* = .02; *I*^2^ = 0%). No increase in serious adverse events was found with inhaled antibiotic treatment (OR, 0.87; 95% CI, 0.69-1.09; *P* = .23; *I*^2^ = 0%). Significantly increased serious adverse effects were found in the AIR-BX trials, with an OR of 10.29 (95% CI, 1.12-94.99; *P* = .04, *I*^2^ = 8%).^29^ Pooled analysis of 20 trials (N = 3,468) showed a nonsignificant increase in adverse events leading to discontinuation with a pooled OR of 1.38 (95% CI, 0.99-1.92; *P* = .06; *I*^2^ = 38%). In the subgroup analysis, a significant increase in discontinuation was found with aztreonam (OR, 3.09; 95% CI, 1.39-6.84; *P* = .006; *I*^2^ = 38%) and aminoglycosides (OR, 2.45; 95% CI, 1.32-4.56; *P* = .005; *I*^2^ = 0%). No increase was seen with fluoroquinolones (OR, 0.90; 95% CI, 0.64-1.26; *P* = .53; *I*^2^ = 0%) or colistin (OR, 0.89; 95% CI, 0.53-1.50; *P* = .67; *I*^2^ = 0%). Adherence was similar between placebo and inhaled antibiotics with an risk ratio of 1.00 (95% CI, 0.97-1.02; *P* = .78), with no heterogeneity (*I*^2^ = 0%).

**Figure 5:**
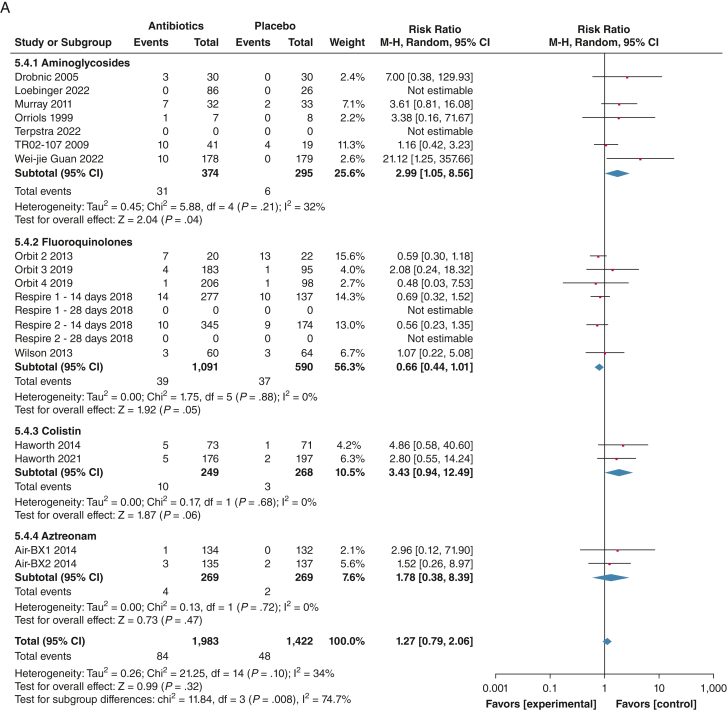

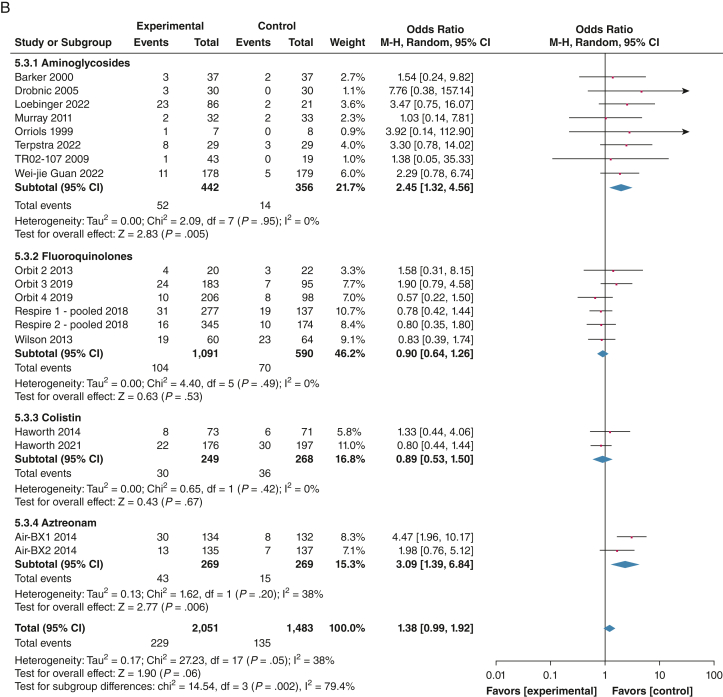
A, B, Forest plots showing treatment-emergent adverse effects leading to discontinuation (A) and bronchospasm events (B). The weight of each study is the percentage of its contribution to the overall effect estimate. Weights of individual studies might not add up to the subtotal or overall weights because of rounding. Risk of bias is represented as low (+), unclear (?), or high (–). Includes safety population. df = degrees of freedom; IV = inverse variance; M-H = Mantel-Haenszel.

With data from 18 trials, bronchospasm was reported in 84 of 1,983 patients (4.2%) in the intervention group and 48 of 1,422 patients (3.4%) in the placebo group. The pooled OR was 1.27 (95% CI, 0.79-2.06; *P* = .32; *I*^2^ = 34%). Subgroup analysis showed increased risk of bronchospasm with aminoglycosides, with 8.3% of patients in the intervention group reporting bronchospasm (RR, 2.99; 95% CI, 1.05-8.56; *P* = .04; *I*^2^ = 32%) (Fig 5).^19^, ^20^, ^21^, ^22^, ^23^, ^24^, ^25^, ^26^, ^27^, ^28^, ^29^, ^30^, ^31^^,^^34^

Mortality was assessed in two new studies, resulting in 15 trials included for this outcome.^19^^,^^27^ Twenty-seven deaths occurred in the intervention group (of 1,794 patients) and 16 deaths occurred in the placebo group (of 1,300 patients). The pooled risk ratio was 1.08 (95% CI, 0.58-2.01; *P* = .85; *I*^2^ = 0).^19^, ^20^, ^21^, ^22^^,^^24^^,^^25^^,^^27^, ^28^, ^29^^,^^34^ Key study results are summarized in Table 2.

**Table 2:** Key Findings of a Meta-Analysis of Inhaled Antibiotics for the Treatment of Adult Patients With Bronchiectasis

Outcomes	Anticipated Absolute Effects (95% CI)^a^		Relative Effect (95% CI)	No. of Participants (Studies)	Certainty of Evidence^b^	Comments
	Risk With Placebo	Risk With Inhaled Antibiotics				
Frequency of exacerbations	93 per 100	73 per 100 (63-84)	RR, 0.78 (0.68-0.90)	2,930 (12 RCTs)	⨁⨁⨁⨁ high^c^	Inhaled antibiotics result in a slight reduction in frequency of exacerbations.
Frequency of severe exacerbations	29 per 100	14 per 100 (9-21)	RR, 0.48 (0.31-0.74)	828 (7 RCTs)	⨁⨁⨁◯ moderate^d^	Inhaled antibiotics likely reduce frequency of severe exacerbations.
Time to first exacerbation	0 per 100	NaN per 100 (— to —)	HR, 0.80 (0.68-0.94)	2,725 (12 RCTs)	⨁⨁◯◯ low^e^^f^	Inhaled antibiotics may increase time to first exacerbation slightly.
Change from baseline QOL-B RSS score (scale, 0-100)	Mean change from baseline QoL-B RSS score was 4.9 points^g^	MD 2.37 points higher (0.44 higher-4.31 higher)	NA	2,315 (11 RCTs)	⨁⨁⨁⨁ high^c^^h^	Inhaled antibiotics probably result in a slight increase in change from baseline QOL-B RSS score, without reaching the minimal clinical important difference of 8 points.
Change from baseline SGRQ score (scale, 0-100)	Mean change from baseline SGRQ score was –0.62 points^i^	MD 3.13 points lower (5.93 lower-0.32 lower)	NA	1,338 (10 RCTs)	⨁◯◯◯ very low^j^^k^^1^^m^^n^	Inhaled antibiotics may reduce or have little to no effect on change from baseline SGRQ score, but the evidence is uncertain.
Isolates with resistant MIC at the end of treatment	9 per 100	17 per 100 (14-21)	Risk ratio, 1.86 (1.51-2.30)	2,619 (17 RCTs)	⨁⨁⨁◯ moderate^n^	Inhaled antibiotics likely result in an increase in isolates with resistant MIC at the end of treatment.
No. of patients reporting TEAE	80 per 100	80 per 100 (75-84)	OR, 0.99 (0.75-1.30)	3,207 (15 RCTs)	⨁⨁⨁◯ moderate^a^^o^	Inhaled antibiotics likely result in little to no difference in number of patients reporting TEAEs.

HR = hazard ratio; MD = mean difference; MIC = minimum inhibitory concentration; NA = not applicable; NaN = not a number; QOL-B = Quality of Life Questionnaire-Bronchiectasis; RCT = randomized controlled trial; RR = rate ratio; RSS = respiratory symptoms scale; SGRQ = St. George Respiratory Questionnaire; TEAE = treatment-emergent adverse event.

a The risk in the intervention group (and its 95% CI) is based on the assumed risk in the comparison group and the relative effect of the intervention (and its 95% CI).

b Grading of Recommendations, Assessment, Development, and Evaluations Working Group grades of evidence: high certainty = we are very confident that the true effect lies close to that of the estimate of the effect; moderate certainty = we are moderately confident in the effect estimate: the true effect is likely to be close to the estimate of the effect, but there is a possibility that it is substantially different; low certainty = our confidence in the effect estimate is limited: the true effect may be substantially different from the estimate of the effect; very low certainty = we have very little confidence in the effect estimate: the true effect is likely to be substantially different from the estimate of effect.

c The heterogeneity can be attributed to the AIR-BX trials. Explanation is provided in the text. *P* value for heterogeneity remains > .05.

d Weight average of studies with high risk of bias is 42.4%, with the rest of the studies being low risk of bias.

e Although most studies are low risk of bias, no significant benefit exists after excluding the studies with high risk of bias.

f Lack of mean or median values in some of the studies because of low no. of events, making it difficult to estimate effect size correctly.

g Based on change from baseline (placebo) in studies of > 48-wk duration.

h Small CI. Although it does not reach the minimal clinical difference (which was defined using the AIR-BX trials), the results are solid with good studies of low risk of bias. For this reason, we decided not to downgrade, because 2.73 points can be relevant in our population.

i Based on change from baseline (placebo) studies with SGRQ as an outcome.

j Unreliable methods in one study.

k Concern in patient and personnel masking and incomplete outcome data.

l Nonoverlapping CI; studies of low risk of bias with opposite results; *P* < .05 for heterogeneity.

m Wide CI overlapping with the minimal clinical difference of 4 points.

n Assymetrical funnel plot, probably exacerbating effects.

o CI includes benefit and harm.

### Bacterial Load and Pathogen Eradication

Eleven studies (N = 2,014) provided data on bacterial load.^22^^,^^23^^,^^26^, ^27^, ^28^, ^29^^,^^31^^,^^32^^,^^34^ Bacterial load was measured by mean reduction in colony-forming units per gram of sputum. The pooled mean reduction was –2.32 colony-forming units/g of sputum (95% CI, –3.06 to –1.58; *P* < .00001), with high heterogeneity (*I*^2^ = 90%).

Bacterial eradication from sputum, defined by the absence of the baseline pathogen on the end-of-treatment sputum sample, was increased significantly with inhaled antibiotic treatment (OR, 3.65; 95% CI, 2.02-6.58; *P* < .0001), with high heterogeneity (*I*^2^ = 75%; N = 2,370). The proportion of patients achieving “eradication” (12 trials) during treatment was 371 of 1,120 patients (33.1%) in the intervention group and 122 of 757 patients (16.1%) in the placebo group.^20^^,^^21^^,^^23^, ^24^, ^25^, ^26^, ^27^, ^28^^,^^30^, ^31^, ^32^, ^33^

### Subgroup Analysis of Studies with *P aeruginosa* and Other Bacterial Pathogens

Few studies reported baseline infection with pathogens other than *P aeruginosa*. In the studies that reported other pathogens in sputum (nine trials; N = 1,710), the prevalence of *P aeruginosa* in the population was still high, with studies ranging from 27% in the BATTLE trial to 82% in the AIR-BX trials.^20^^,^^21^^,^^24^^,^^29^^,^^31^^,^^33^ The proportion of patients with at least one exacerbation was reduced significantly in this subgroup, with 375 of 1,008 patients (37.2%) treated with inhaled antibiotics having at least one exacerbation compared with 296 of 702 patients (42.1%) in the placebo group (RR, 0.84; 95% CI, 0.72-0.98; *P* = .03; *I*^2^ = 47%). Pooled analysis showed a nonsignificant reduction in time to first exacerbation (HR, 0.82; 95% CI, 0.65-1.05; *P* = .11; *I*^2^ = 52%) and exacerbation rate (RR, 0.83; 95% CI, 0.68-1.02; *P* = .07; *I*^2^ = 46%). Analysis without the AIR-BX studies (five trials; N = 989) showed significant improvement in frequency of exacerbations (RR, 0.77; 95% CI, 0.69-0.86; *P* = .003; *I*^2^ = 22%) and time to first exacerbation (RR, 0.71; 95% CI, 0.59-0.86; *P* = .0004, *I*^2^ = 0%). These results are derived mainly from the RESPIRE trials.^20^^,^^21^

Eradication was accomplished in 32.1% of patients in the intervention population and 20.1% of patients in the placebo population. Pooled analysis showed an OR of 3.04 (95% CI, 1.32-6.98; *P* = .009; *I*^2^ = 80%), with data obtained from seven trials (N = 1,135).^20^^,^^21^^,^^24^^,^^31^^,^^33^ Resistance was reported in nine trials, with an RR of 2.27 (95% CI, 1.70-3.03; *P* < .00001; *I*^2^ = 0%).^20^^,^^21^^,^^24^^,^^29^^,^^31^^,^^33^

### Maintenance Therapy

In a sensitivity analysis focusing on studies with therapy durations of at least 3 months, we excluded Barker et al,^32^ which had a 6-week treatment period. No significant changes were observed in antibiotic resistance (RR, 1.87; 95% CI, 1.50-2.34; *P* < .00001; *I*^2^ = 11%), TEAEs (RR, 0.99; 95% CI, 0.74-1.32; *P* = .93; *I*^2^ = 47%), number of adverse events leading to discontinuation (RR, 1.38; 95% CI, 0.98-1.95; *P* = .06; *I*^2^ = 41%), adherence (RR, 1.00; 95% CI, 0.98-1.02; *P* = .85; *I*^2^ = 4%), bacterial load (RR, –2.07; 95% CI, –2.70 to –1.43; *P* < .00001; *I*^2^ = 86%), or eradication (RR, 3.30; 95% CI, 1.85-5.88; *P* < .0001; *I*^2^ = 74%).

### Risk of Bias

**Supplementary Figure 1:**
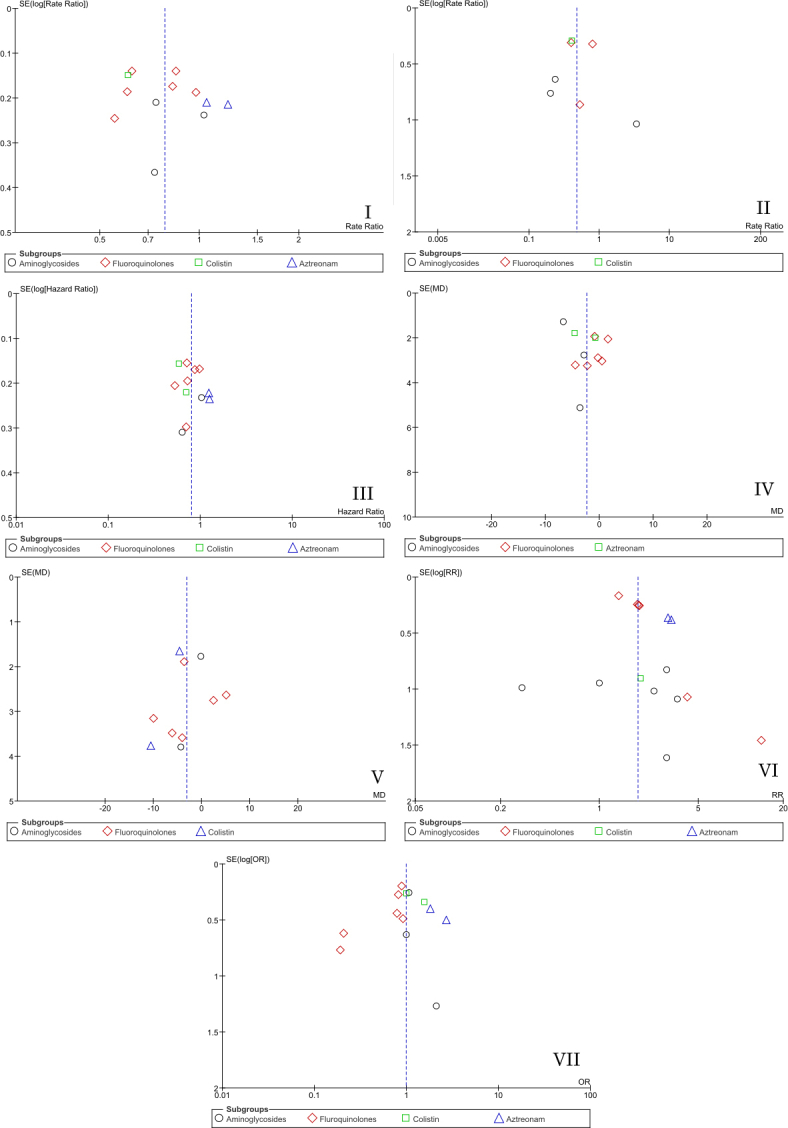


**Supplementary Figure 2:**
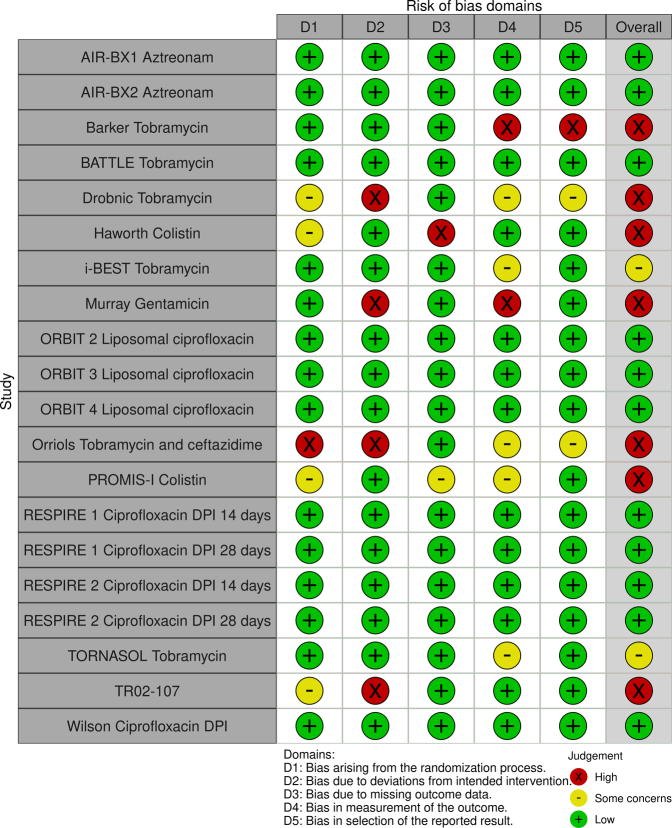


We assessed the risk of bias using the Cochrane risk of bias tool, RoB 2.^35^ A high risk of bias was reported in five studies included in the previous meta-analysis.^24^^,^^25^^,^^28^^,^^30^^,^^32^ Regarding the new studies, three showed a low risk or unclear risk of bias,^26^^,^^27^^,^^33^ and one study was considered to show a high risk of bias (e-Table 3, e-Fig 1 [I-VII], and e-Fig 2).^19^

## Discussion

This updated systematic review and meta-analysis provides novel information about the safety and efficacy of inhaled antibiotics in patients with bronchiectasis, with a focus in long-term maintenance therapy, with all the studies except one^32^ having a duration of > 3 mo. We show that inhaled antibiotics are associated with a significant reduction in pulmonary exacerbations, with a pooled estimate of a 21% reduction. A much larger effect was observed on severe exacerbations requiring hospitalization or IV antibiotics at 52%. No minimal clinically important difference is reported for exacerbation reduction in bronchiectasis and attempts to derive such values have been controversial in other fields such as COPD.^36^ Nevertheless, in other diseases reductions of > 20% are regarded as highly clinically relevant.^37^ We conclude that inhaled antibiotics are associated with a clinically relevant reduction in exacerbations in diverse populations.

A striking finding of our analysis was that despite frequent discussion of the inconsistent results between inhaled antibiotic trials,^38^^,^^39^ the results for exacerbations showed minimal heterogeneity and were remarkably consistent despite multiple differences in design, patient population, duration, and the antibiotic used. After exclusion of the AIR-BX studies, a set of studies that were notably different because they used no enrichment for patients with frequent exacerbations, no requirement for chronic infection at baseline, and a short follow-up duration, no significant heterogeneity in results between studies was found.^29^ Our analysis suggests that the true treatment effect lies somewhere between a 16% and 35% reduction in exacerbations. The inconsistent results of various trials simply may reflect that individual studies are powered frequently on the basis of much larger effects. For example, we found that inhaled antibiotics prolonged time to first exacerbation by 20%, but the RESPIRE trials were powered on a median increase of 67%.^40^ Therefore, although the frequently cited heterogeneity of the disease may be part of the explanation for the failure to achieve two replicate-positive randomized clinical trials in bronchiectasis for inhaled antibiotics, a failure to power trials properly for an average treatment effect of approximately 20% reduction in exacerbation frequency also may be a factor.

A key novel finding of our analysis is that inhaled antibiotics significantly improve symptoms and quality of life, contradicting the results of the previous meta-analysis and some of the large phase 3 trials. We observed small but significant improvements in both the Quality of Life Questionnaire-Bronchiectasis and St. George Respiratory Questionnaire scores. The consistency in these results between two tools applied in different trials makes it more likely that these estimates are true reflections of the underlying treatment effect. Although neither difference is greater than the minimum clinically important difference on average, it is likely that some patients will experience a clinically meaningful change in symptoms. A post hoc analysis of the ORBIT trials identified a significantly higher number of patients experiencing an 8-point improvement in the Quality of Life Questionnaire-Bronchiectasis in the treatment vs the placebo group.^41^ A recent study found that patients experiencing a symptom improvement are not necessarily the same patients who experience a reduction in exacerbations.^42^ A high degree of heterogeneity in symptoms responses was found between studies, and further research is needed to understand why some populations experience a large symptom improvement and many do not. Patients are more likely to experience a symptom improvement if they show symptoms including cough and sputum production, because antibiotics primarily improve these symptoms.^14^ In support of this, we found that inhaled antibiotics reduce sputum volume. The magnitude of the differences observed in our analysis suggest that inhaled antibiotics will not be used primarily to reduce symptoms, but that patients being prescribed inhaled antibiotics to reduce exacerbations can be told that some patients also will experience a significant improvement in symptoms.

Safety of inhaled antibiotics is a key consideration, and in this regard, our results are reassuring. Adverse events were not increased significantly. Bronchospasm, which is a concern with inhaled antibiotics, also was not increased significantly except for with aminoglycosides. Withdrawals were more likely with aminoglycosides and aztreonam. Our data suggest that aminoglycosides are more likely to cause bronchospasm and discontinuation of treatment than other antibiotic classes.

An important subanalysis of our data suggested that the magnitude of benefits observed in populations including both *P aeruginosa* and non-*P aeruginosa* infections are comparable, and this is consistent with subgroup analysis data from mixed studies such as the RESPIRE trial, where no differences in response were observed between the groups.^20^^,^^21^ Current ERS guidelines suggest choosing inhaled antibiotics for *P aeruginosa* infections and oral antibiotics for non-*P aeruginosa* infections. Since 2017, macrolides have been shown to have efficacy in reducing exacerbations in patients infected with *P aeruginosa*, albeit in smaller numbers than have been studied in the inhaled antibiotic trials.^7^ Our data suggest that both macrolides and inhaled antibiotics are effective in both populations, and the choice of treatment may come down to the balance of risks and benefits of each drug for the individual patient, rather than the baseline pathogen.

## Interpretation

In summary, this updated systematic review and meta-analysis offers a number of important observations, including a clinically relevant reduction in exacerbations and severe exacerbations with inhaled antibiotics, significant improvements in symptoms and quality of life, and reassuring safety and efficacy across the spectrum of respiratory pathogens.

## Funding/Support

This work was supported by the 10.13039/501100010767Innovative Medicines Initiative and European Federation of Pharmaceutical Industries and Association companies under the European Commission-funded project iABC [Grant 115721] and by the European Respiratory Society through the European Multicentre Bronchiectasis Audit and Research Collaboration-3 (EMBARC3) consortium. EMBARC3 is supported by project partners Armata, 10.13039/100004325AstraZeneca, Boehringer-Ingelheim, 10.13039/100019719Chiesi, 10.13039/100008322CSL Behring, 10.13039/501100016387Grifols, 10.13039/100020483Insmed, 10.13039/100005565Janssen, 10.13039/100012357Lifearc, and Zambon. J. D. C. is supported by the Asthma and Lung UK Chair of Respiratory Research and a Scottish Senior Fellowship from the Chief Scientist Office (United Kingdom).

## Financial/Nonfinancial Disclosures

The authors have reported to *CHEST* the following: H. C. reports grants or contracts from the Korean Ministry of Education Basic Science Research Program [Grant 2021R1I1A3052416]; consulting fees from Boryung Pharmaceutical Co., Ltd.; and payment or honoraria for lectures, presentations, speakers bureaus, manuscript writing, or educational events from Boryung Pharmaceutical Co., Ltd. C. S. H. reports consulting fees from 30 Technology, Aradigm, CSL Behring, Chiesi, Gilead, Grifols, GSK, Insmed, Janssen, LifeArc, Meiji, Mylan, Novartis, Pneumagen, Shionogi, Teva, Vertex, and Zambon (personal fees); payment or honoraria for lectures, presentations, speakers bureaus, manuscript writing, or educational events from Chiesi, Insmed; and payment for expert testimony from Zambon. J. D. C. has received research grants from AstraZeneca, Boehringer Ingelheim, GlaxoSmithKline, Gilead Sciences, Grifols, Novartis, Insmed, and Trudell and has received consultancy or speaker fees from Antabio, AstraZeneca, Boehringer Ingelheim, Chiesi, GlaxoSmithKline, Insmed, Janssen, Novartis, Pfizer, Trudell, and Zambon. None declared (R. C.).

## Supplementary Data

e-Online Data

e-Online Data
