# Supplementary material for: The Efficacy and Safety of Inhaled Antibiotics for the Treatment of Bronchiectasis in Adults: Updated Systematic Review and Meta-Analysis
Source: Chest. 2024 Feb 2;166(1):61–80. doi: 10.1016/j.chest.2024.01.045 (PMC11251083; doi:10.1016/j.chest.2024.01.045)
Supplement: e-Online Data [file mmc1.docx]

**ONLINE SUPPLEMENTARY METHODS**

**Full search strategy**

*Electronic searches*

1. Cochrane ultrasensitive search strategy in MEDLINE/PUBMED as follows: (((((randomized controlled trial[pt] OR controlled clinical trial[pt] OR randomized[tiab] OR placebo[tiab] OR clinical trials as topic[MeSH:noexp] OR randomly[tiab] OR trial[ti] NOT (animals[mh] NOT humans [mh])))) AND "bronchiectasis" AND "antibiotics" AND ("2019/01"[Date – Publication] : "2022/12"[Date - Publication])).

2. EMBASE as follows: ((single blind procedure or Antibiotic or Ciprofloxacin or Tobramycin or allocat *or double blind procedure or Aztreonam or Placebo or Nebuli* or Inhale* or crossover or cross over) and Bronchiectasis).af. limit to (human and (randomized controlled trial or controlled clinical trial or phase 2 clinical trial or phase 3 clinical trial or phase 4 clinical trial) and yr="2019 - 2022")

3. Cochrane central database as follows (custom data range: 21/01/2019 to 13/12/2022): a. #1 Bronchiectasis

b. #2 MeSH descriptor: [Bronchiectasis] explode all trees

c. #3 (Bronchiectases):ti,ab,kw OR (bronchial NEXT disease*):ti,ab,kw (Word variations have been searched)

d. #4 #1 or #2 or #3

e. #5 MeSH descriptor: [Anti-Bacterial Agents] explode all trees

f. #6 MeSH descriptor: [Tobramycin] explode all trees

g. #7 MeSH descriptor: [Aztreonam] explode all trees

h. #8 MeSH descriptor: [Ciprofloxacin] explode all trees

i. #9 MeSH descriptor: [Health Care Rationing] explode all trees

j. #10 MeSH descriptor: [Single-Blind Method] explode all trees

k. #11 MeSH descriptor: [Double-Blind Method] explode all trees

l. #12 MeSH descriptor: [Cross-Over Studies] explode all trees

m. #13 MeSH descriptor: [Placebos] explode all trees

n. #14 MeSH descriptor: [Nebulizers and Vaporizers] explode all trees

o. #15 MeSH descriptor: [Inhalation] explode all trees

p. #16 #5 OR #6 OR #7 OR #8 OR #9 OR #10 OR #11 OR #12 OR #13 OR #14 OR #15 q. #17 #4 AND #16

*Deciding on how to approach the data*

The decision to conduct a traditional meta-analysis was driven by the ample number of studies (N=20) and participants (N=3468), which ensures the avoidance of type I errors commonly associated with smaller meta-analyses, precluding the need for alternative methods such as sequential meta-analysis.

*Search strategy and selection criteria*

When results were not reported in a format suitable for meta-analysis, we used the recommended methods from the Cochrane Handbook to extract or estimate effects. This included trying to contact authors and pharmaceutical company sponsors to request unpublished data, reviewing abstract books or presentations provided at International Conferences, reviewing regulatory agents' data (FDA, EMA), reviewing supplemental data and browsing the clinicaltrials.gov database for published trial results.

When neither of these options provided the data endpoints, we used the recommended methods for estimating data by using formulae to extract SEs, SDs, or CIs from the presented data, conversion of medians to estimated mean and SD from the Cochrane Handbook.

When data had been estimated, sensitivity analyses excluding such data were performed to check the influence of any assumptions on the reported pooled effects.

When trials had two intervention groups and a matched placebo for each group the effect estimates versus matching placebo were reported. When these results were not available, data from the two intervention groups were pooled and matched against the placebo group. ´

For data requiring transformation, we selected the Inverse Variance (IV) method as the primary approach, given its ability to assimilate standard errors and evaluate heterogeneity. For outcomes with untransformed binary data and low event rates, we employed the Mantel-Haenszel (M-H) method, given its robustness in handling sparse data.

Data transformation using RevMan underwent subsequent validation using with R version 4.2.2.

*Presenting the data*

For dichotomous outcomes, data are presented as pooled risk ratios or odds ratios (ORs) and 95% CIs. Continuous variables are presented as mean differences with 95% CI. Count data outcomes were extracted and analysed as rate data if information about the total number of events and amount of person-time at risk in each group was present. Effect estimates were pooled by the inverse of their variance and are presented as pooled effect estimates (hazard ratios or rate ratios) with corresponding 95% CIs. All analyses used random effects meta-analysis using the method of DerSimonian and Laird because of the heterogeneity of study designs. The threshold for significance for p values was 0.05.

*Thresholds used to interpret the size of effects*

When outcomes included patient-reported outcome measures (PROMs), the corresponding minimally important difference obtained by anchor methods were used to interpret the size of the effect. Mean difference absolute values without MID are presented alongside baseline values for interpretation (if available).

*Dealing with missing data*

The authors evaluated the amount and causes of dropouts and withdrawals in both groups and acted per the guidelines provided by the Cochrane handbook collaboration.

*Dealing with unclear information*

The authors discussed unclear information to determine whether it was suitable for inclusion in the meta-analysis. If the outcomes were deemed unacceptable for analysis, they were not included.

*Risk of bias assessment*

We used the Cochrane Risk-of-bias tool (RoB 2), as per Cochrane suggestions on updated meta-analyses.

*Between-study variance (Tau²)*

Tau is automatically calculated in revman using the two‐step DerSimonian and Laird method (Veroniki et al., 2016).

*Dealing with Zero Cells or no events in both groups*

Using the RevMan software, zero cell events were automatically adjusted with a fixed value (0.5). Nonetheless we performed a Haldane-Anscombe correction using R version 4.2.2 for better data comparison and transparency, while also estimating the heterogeneity variance (tau^2) using the REML approach.

*Results for the Haldane-Anscombe correction and Tau²*

Isolates with resistant MIC at the end of treatment: Tau² = 0.00; Chi = 8.6442, df = 16, I²= 0.00%, OR = 1.8422 [1.4596 to 2.3251]; Number of patients reporting TEAE: Tau² = <0.0001; Chi = 20.55, df = 12, I²= 41.6%, OR = 0.9893 [0.8168 to 1.1982]; Number of patients with serious TEAE: Tau² = <0.0001; Chi = 10.46, df = 11, I²= 0.0%, OR = 0.8733 [0.6933 to 1.0999]; Number of AE leading to discontinuation: Tau² = 0.2011; Chi = 26.35, df = 17, I²= 35.5%, OR = 1.2089 [0.9572 to 1.8983]; Number of patients reporting bronchospasm: Tau² = 0.2733; Chi = 19.30, df = 15, I²= 22.3%, OR = 1.2089 [0.7329 to 1.9939]; All cause mortality: Tau² = 0; Chi = 4.67, df = 12, I²= 0.0%, OR = 1.0576 [0.5766 to 1.9400]; Eradication: Tau² = 0.6681; Chi = 38.80, df = 11, I²= 71.6%, OR = 3.6104 [1.9791 to 6.5864]
