# Supplementary material for: The Efficacy and Safety of Inhaled Antibiotics for the Treatment of Bronchiectasis in Adults: Updated Systematic Review and Meta-Analysis
Source: Chest. 2024 Feb 2;166(1):61–80. doi: 10.1016/j.chest.2024.01.045 (PMC11251083; doi:10.1016/j.chest.2024.01.045)
Supplement: e-Online Data [file mmc2.docx]

| **Wilson Ciprofloxacin DPI** | Increases in minimum inhibitory concentration (MIC) to 4 mg/L (a level defined as clinically relevant for systemic therapy with ciprofloxacin), which were considered to be probably or possibly related to the administration of study drug. |
| --- | --- |
| **RESPIRE 1**  **Ciprofloxacin DPI** | The number of patients with the development of pathogens with elevated MICs (resistant by systemic breakpoints) from pre-treatment to any time point during the study. |
| **RESPIRE 2**  **Ciprofloxacin DPI** |  |
| **ORBIT 2**  **Liposomal ciprofloxacin** | The identification of *P. aeruginosa* isolates with lowered categorical susceptibility to ciproﬂoxacin, according to the CLSI guideline breakpoints, at any time during the study (we reported only those classified as resistant, although the original manuscript also reported those with intermediate  susceptibility). |
| **ORBIT 3**  **Liposomal ciprofloxacin** | Data are presented in the manuscript as percentage of isolates with two-fold or greater increase in MIC during the study. Data obtained from FDA briefing document with number of isolates with MIC above the systemic breakpoint (4 mg/L) at any time during treatment in those susceptible at baseline. The latter data was used to be concordant with data obtained from other trials. |
| **ORBIT 4**  **Liposomal ciprofloxacin** |  |
| **AIR-BX1**  **Aztreonam** | Increases of four-fold or higher in the MIC of aztreonam for target Gram-negative organisms at 12 weeks. |
| **AIR-BX2**  **Aztreonam** |  |
| **Drobnic**  **Tobramycin** | Tobramycin-resistant *P. aeruginosa* isolated during the study using systemic breakpoints |
| **Barker Tobramycin** | Isolation of tobramycin-resistant *P. aeruginosa* at the last visit. |
| **Orriols Tobramycin and**  **ceftazidime** | Emergent antibiotic-resistant bacteria (presumed to be isolation of resistant *P. aeruginosa* at any point during the study). |
| **Murray Gentamicin** | Gentamicin susceptibility testing was performed for all isolates of *P. aeruginosa* and Gram-negative enteric bacteria at Months 0, 12, and 15, according to the CLSI guidelines.Gentamicin resistance was defined by systemic breakpoints. |
| **Haworth Colistin** | Isolation of colistin resistant organisms defined by MIC >4mg/L. |

Abbreviations: DPI, dry powder for inhalation; CLSI, Clinical and Laboratory Standards Institute.

| **BATTLE**  **Tobramycin** | Isolation of tobramycin resistant *P. aeruginosa* at the end of the study. |
| --- | --- |
| **i-BEST**  **Tobramycin** | Four-fold or greater increase in TIP MIC compared to baseline. |
| **PROMIS-I**  **Colistin** | Isolation of colistin-resistant *P. aeruginosa* at the last visit. |
| **TORNASOL**  **Tobramycin** | MIC being 16 or greater for tobramycin. |

Abbreviations: TIP, Tobramycin Inhalation Powder; MIC, Minimum inhibitory concentration.

|  | **AIR-BX1 Aztreonam** | **AIR-BX2 Aztreonam** | **Barker Tobramycin** | **BATTLE Tobramycin** | **Drobnic Tobramycin** | **Haworth Colistin** | **i-BEST Tobramycin** | **Murray Gentamicin** | **ORBIT 2 Liposomal ciprofloxacin** | **ORBIT 3 Liposomal ciprofloxacin** | **ORBIT 4 Liposomal ciprofloxacin** | **Orriols Tobramycin and ceftazidime** | **PROMIS-I Colistin** | **RESPIRE 1 Ciprofloxacin DPI 14 days** | **RESPIRE 1 Ciprofloxacin DPI 28 days** | **RESPIRE 2 Ciprofloxacin DPI 14 days** | **RESPIRE 2 Ciprofloxacin DPI 28 days** | **TORNASOL Tobramycin** | **TR02-107** | **Wilson Ciprofloxacin DPI** |
| --- | --- | --- | --- | --- | --- | --- | --- | --- | --- | --- | --- | --- | --- | --- | --- | --- | --- | --- | --- | --- |
| **Domain 1: Risk of bias arising from the randomization process** | Low risk | Low risk | Low risk | Low risk | Some concerns | Some concerns | Low risk | Low risk | Low risk | Low risk | Low risk | High risk | Some concerns | Low risk | Low risk | Low risk | Low risk | Low risk | Some concerns | Low risk |
| **1.1 Was the allocation sequence random?** | Y | Y | Y | Y | NI | PY | Y | PY | Y | Y | Y | NI | PY | Y | Y | Y | Y | Y | NI | Y |
| **1.2 Was the allocation sequence concealed until participants were enrolled and assigned to interventions?** | Y | Y | Y | Y | NI | Y | Y | PY | Y | Y | Y | N | NI | Y | Y | Y | Y | PY | NI | PY |
| **1.3 Did baseline differences between intervention groups suggest a problem with the randomization process?** | N | N | N | N | PN | N | N | PN | N | N | N |  | PN | PN | PN | PN | PN | N | NI | PN |
| Domain 2: Risk of bias due to deviations from the intended interventions (effect of assignment to intervention) | Low risk | Low risk | Low risk | Low risk | High risk | Low risk | Low risk | High risk | Low risk | Low risk | Low risk | High risk | Low risk | Low risk | Low risk | Low risk | Low risk | Low risk | High risk | Low risk |
| **2.1. Were participants aware of their assigned intervention during the trial?** | N | N | N | N | PY | N | N | Y | N | N | N | Y | PN | N | N | N | N | N | NI | N |
| **2.2. Were carers and people delivering the interventions aware of participants' assigned intervention during the trial?** | N | N | N | N | PY | N | N |  | N | N | N | Y | PN | N | N | N | N | PN | NI | N |
| **2.3. If Y/PY/NI to 2.1 or 2.2: Were there deviations from the intended intervention that arose because of the trial context?** |  |  |  | PN | PY |  |  |  |  |  |  | PY |  |  |  |  |  |  | NI |  |
| **2.4 If Y/PY to 2.3: Were these deviations likely to have affected the outcome?** |  |  |  |  | PY |  |  |  |  |  |  | PY |  |  |  |  |  |  |  |  |
| **2.5. If Y/PY/NI to 2.4: Were these deviations from intended intervention balanced between groups?** |  |  |  |  | PN |  |  |  |  |  |  | NI |  |  |  |  |  |  |  |  |
| **2.6 Was an appropriate analysis used to estimate the effect of assignment to intervention?** | Y | Y | Y | Y | PN | Y | Y |  | Y | Y | Y | N | PY | Y | Y | Y | Y | Y | NI | Y |
| **2.7 If N/PN/NI to 2.6: Was there potential for a substantial impact (on the result) of the failure to analyse participants in the group to which they were randomized?** |  |  |  |  | PN |  |  |  |  |  |  |  |  |  |  |  |  |  | NI |  |
| Domain 3: Risk of bias due to missing outcome data | Low risk | Low risk | Low risk | Low risk | Low risk | High risk | Low risk | Low risk | Low risk | Low risk | Low risk | Low risk | Some concerns | Low risk | Low risk | Low risk | Low risk | Low risk | Low risk | Low risk |
| **3.1 Were data for this outcome available for all, or nearly all, participants randomized?** | Y | Y | Y | Y | Y | N | PY | PY | Y | Y | Y | Y | PN | Y | Y | Y | Y | Y | Y | Y |
| **3.2 If N/PN/NI to 3.1: Is there evidence that the result was not biased by missing outcome data?** |  |  |  |  |  |  |  |  |  |  |  |  | PN |  |  |  |  |  |  |  |
| **3.3 If N/PN to 3.2: Could missingness in the outcome depend on its true value?** |  |  |  |  |  |  |  |  |  |  |  |  | PY |  |  |  |  |  |  |  |
| **3.4 If Y/PY/NI to 3.3: Is it likely that missingness in the outcome depended on its true value?** |  |  |  |  |  |  |  |  |  |  |  |  | PN |  |  |  |  |  |  |  |
| Domain 4: Risk of bias in measurement of the outcome | Low risk | Low risk | High risk | Low risk | Some concerns | Low risk | Some concerns | High risk | Low risk | Low risk | Low risk | Some concerns | Some concerns | Low risk | Low risk | Low risk | Low risk | Some concerns | Low risk | Low risk |
| **4.1 Was the method of measuring the outcome inappropriate?** | PN | PN | PY | PN | N | N | PN | PY | N | N | N | N | N | PN | PN | PN | PN | PN | PN | PN |
| **4.2 Could measurement or ascertainment of the outcome have differed between intervention groups?** | PN | PN | PN | N | N | PN | PN |  | N | N | N | PN | PN | PN | PN | PN | PN | PY | PN | PN |
| **4.3 If N/PN/NI to 4.1 and 4.2: Were outcome assessors aware of the intervention received by study participants?** | N | N | N | N | PY | PN | NI |  | N | N | N | Y | NI | N | N | N | N | NI | NI | PN |
| **4.4 If Y/PY/NI to 4.3: Could assessment of the outcome have been influenced by knowledge of intervention received?** |  |  |  | N | PY |  | PY |  |  |  |  | PY | PN |  |  |  |  | PN | PN |  |
| **4.5 If Y/PY/NI to 4.4: Is it likely that assessment of the outcome was influenced by knowledge of intervention received?** |  |  |  | PN | PN |  | PN |  |  |  |  | PN | PN |  |  |  |  |  |  |  |
| Domain 5: Risk of bias in selection of the reported result | Low risk | Low risk | High risk | Low risk | Some concerns | Low risk | Low risk | Low risk | Low risk | Low risk | Low risk | Some concerns | Low risk | Low risk | Low risk | Low risk | Low risk | Low risk | Low risk | Low risk |
| **5.1 Were the data that produced this result analysed in accordance with a pre-specified analysis plan that was finalized before unblinded outcome data were available for analysis?** | Y | Y |  | Y | N | Y | Y | Y | Y | Y | Y | PN | Y | Y | Y | Y | Y | Y | PY | Y |
| **5.2…. multiple eligible outcome measurements (e.g. scales, definitions, time points) within the outcome domain?** | N | N | PY | N | PN | N | N | N | N | N | N | PY | Y | N | N | N | N | N | PN | N |
| **5.3 … multiple eligible analyses of the data?** | N | N |  | N | PN | N | N | N | N | N | N | N | N | N | N | N | N | N | PN | N |
